# Expression of the Arabidopsis Sigma Factor *SIG5* Is Photoreceptor and Photosynthesis Controlled

**DOI:** 10.3390/plants3030359

**Published:** 2014-08-18

**Authors:** Marina Mellenthin, Ulrike Ellersiek, Anna Börger, Margarete Baier

**Affiliations:** 1 Plant Sciences, Heinrich-Heine-Universität, Universitätsstraße 1, Düsseldorf 40225, Germany; E-Mails: marina.mellenthin@uni-duesseldorf.de (M.M.); ulrike.ellersiek@uni-duesseldorf.de (U.E.); 2 Plant Physiology, Freie Universität Berlin, Königin-Luise-Straße 12-16, Berlin 14195, Germany; E-Mail: anna.boerger@fu-berlin.de

**Keywords:** *SIG5*, sigma factor, phytochrome, cryptochrome, photosynthesis, plastoquinone, photosystem II, retrograde signal

## Abstract

Two collections of Arabidopsis GAL4 enhancer trap lines were screened for light-intensity dependent reporter gene activation. Line N9313 was isolated for its strong light-intensity regulation. The T-DNA element trapped distant enhancers of the *SIG5* promoter, which drives expression of a sigma factor involved in regulation of chloroplast genes for photosystem II core proteins. The T-DNA insertion 715 bp upstream of the transcription initiation site splits the promoter in a distal and proximal part. Both parts are sensitive to blue and red light and depend on photosynthetic electron transport activity between photosystem II and the plastoquinone pool. The mainblue-light sensitivity is localized within a 196-bp sequence (–887 to –691 bp) in the proximal promoter region It is preferentially CRY1 and PHYB controlled. Type-I and type-II phytochromes mediate red-light sensitivity via various promoter elements spread over the proximal and distal upstream region. This work characterizes *SIG5* as an anterograde control factor of chloroplast gene expression, which is controlled by chloroplast signals in a retrograde manner.

## 1. Introduction

Light provides the main source of energy for plant life. Furthermore, light is a key regulator of plant development and metabolism. Many light-responses include gene expression regulation [1,2]. *Cis*-acting regulatory elements control the combination, spacing and relative orientation of transcription factors, which in turn make the promoters sensitive to light intensities and light qualities in a gene-specific manner [2,3]. Most light responsive elements (LREs) have been identified by characterization of the promoter regions of photosynthesis-associated genes [4]. Motifs, such as G-boxes (CACGTG; [5]), Z-boxes (ATACGTGT; [6]), I-boxes (GATAAGR; [7]) and GATA-motifs (GATA; [8]) have mainly been identified in close vicinity (within 300 bp upstream) to the transcription initiation site. In the core-promoters, specific TATA-boxes/Inr-elements increase the light sensitivity [9]. More distant elements were identified by foot-printing and gel-retardation assays in screens for binding motifs of light-responsive transcription factors [1,10]. Despite these intensive gene-specific studies, knowledge on light-regulation of gene expression is still fragmentary [11,12].

Light regulates organellar and nuclear gene expression [13,14]. It controls germination, photomorphogenesis and plant development [15,16]. Light signals are sensed by photoreceptors, such as phytochromes, cryptochromes and phototropins, and the photosystems [17,18]. Some light signals are directly targeted to promoter element-bound transcription factors [10], others are interwoven in developmental and stress-regulated signaling cascades [14,19]. Light, besides water and nutrients, is the most important environmental regulator in plants.

To screen for light-regulated enhancers, irrespective of the function of the regulated gene and the position relative to the transcription initiation site, we analyzed *Arabidopsis thaliana* *GAL4-GFP* enhancer trap lines [20,21] for light-responsiveness. If the enhancer trap T-DNA is inserted in the vicinity of an enhancer element, the *GAL4-VP16* transcriptional activator is expressed. The GAL4-factor subsequently induces expression of an ER-targeted *GFP* (green fluorescent protein) (*mGFP5ER)* which can be monitored non-invasively [20,21].

Previously, enhancer trap lines have been used to identify regulatory sequences controlling developmental and organ-specific expression patterns, e.g., senescence [22], stomatal guard cell development [21] or lateral root development [23]. Here, we used *GAL4 GFP* enhancer trap populations (GAL4 *GFP* ET) to screen for light-responsive promoter elements irrespective of their location relative to the transcription start site. Lines with strong *GFP* expression in mesophyll cells were isolated and sub-selected for responsiveness to light intensity variation. A trap insertion in the distal part of the *SIG5* upstream region gave novel insights into the light-regulation of a nuclear-encoded regulator of chloroplast gene expression. *SIG5* (At5g24120) encodes the sigma factor, which activates expression of the D1 and D2 protein genes (*psbA* and *psbD*) inside chloroplasts [24]. It is required for light dependent regulation during the day [25] and, therefore, an essential factor in the nuclear control of chloroplast function. Here, we show that *SIG5* is regulated by the combined action of multiple blue- and red-light sensitive elements spread over the full promoter and by photosynthetic electron transport.

## 2. Results

### 2.1. Selection of Enhancer-Trap Lines

To identify distal promoter elements involved in light-regulation of gene expression in mesophyll cells, two collections of light grown *GAL4 GFP* enhancer trap lines [20,21] were screened by fluorescence microscopy for mesophyll activity of the enhancer at an age of 7–13 days. At this age, plants are fully shifted from heterotrophic lipid consumption to photoautotrophy and the first true leaves develop [26].

**Figure 1:**
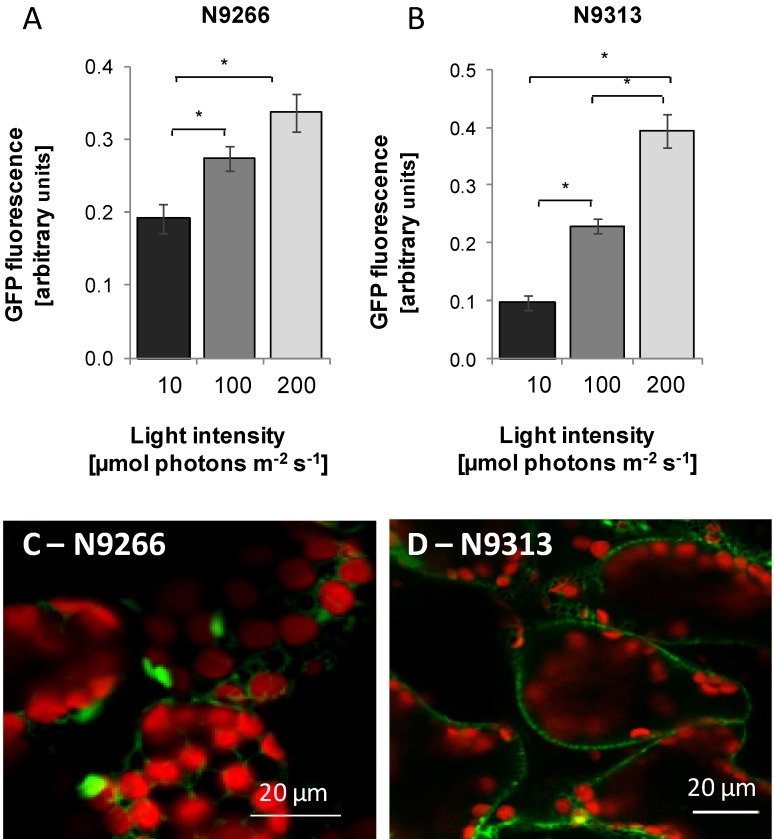
*GFP* fluorescence of *GAL4-GFP* enhancer trap lines. (**A** and **B**): Light-intensity regulation in N9266 and N9313. Seedlings were grown for 10 days in 10, 100 and 200 µmol·photons·m^−2^·s^−1^ white light. One-way ANOVA was performed comparing all groups with Bonferroni’s post-test. Statistical significance of difference is indicated as asterisks above bars (*p* < 0.05); (**C** and **D**): Transverse confocal section of spongy mesophyll of cotyledons. The fluorescence of ER-targeted *GFP* (m*GFP*5ER) is presented in green, Chlorophyll fluorescence in red.

For each selected line, twenty four 10 day old seedlings were screened for light-intensity regulation by comparison of the *GFP*-activity at 10, 100 or 200 µmol·photons·m^−2^·s^−1^ white light using a top reader fluorometer. The crude data were corrected by subtraction of the background fluorescence of wildtype C24 plants, which is caused e.g., by cell wall fluorescence. In several enhancer trap lines the *GFP* fluorescence positively correlated with the growth light intensity. The strongest gradual light-intensity regulation was observed for the lines N9266 and N9313 (Figure 1A,B). Smaller leaves in low light and larger ones at higher light intensities can mock light intensity regulation, while longer hypocotyls would partly mask it. Corrections were performed by standardization of the *GFP* activity on the leaf area. In N9266 and N9313, light intensity regulation was pronounced confirming light-intensity dependent regulation.

### 2.2. Identification of Enhancer Trapped Sequences and T-DNA Numbers

**Figure 2:**
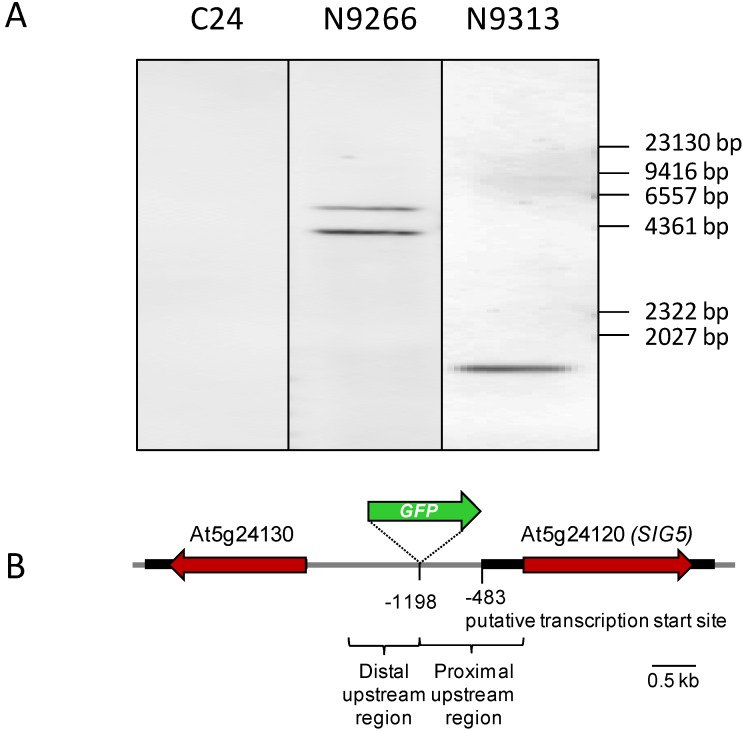
(**A**) Determination of the T-DNA insertion numbers by Southern blot hybridization with a DIG-labeled *GAL4* specific probe. Numbers of DIG-labeled fragments indicate numbers of T-DNA insertions in the different enhancer trap lines; (**B**) T-DNA insertion site and direction of the reporter element (*GFP*, green arrow) and the coding sequences of the flanking genes (red arrows) in the line N9313.

For line N9266, hybridization of Southern-Blots with probes against the GAL4 element gave two bands (Figure 2A). Since DNA was digested with *Bgl*I, which does not cut within the GAL4 element, two bands indicate two T-DNA insertions. In contrast, the hybridization pattern of line N9313 showed that the *GFP* fluorescence results from a single T-DNA insertion (Figure 2A).

To identify the T-DNA insertion site of N9313, 3-step thermal asymmetric interlaced PCR (TAIL-PCR; [27]) was performed with genomic DNA. The tertiary TAIL-PCR product was cloned into pJET1.2/blunt cloning vector (Fermentas, St. Leon-Rot, Germany) and sequenced using a vector specific primer. The sequencing product contained at one end the 35S-minimal promoter demonstrating that it is specific for the enhancer trap insertion site. Comparison of the sequence upstream of the 35S-minimal promoter with *Arabidopsis thaliana* genome sequence data revealed that the T-DNA of line N9313 is inserted 1198 bp upstream of the coding sequence of *SIG5* (At5g24120) (Figure 2B). The *SIG5* gene encodes a sigma factor which is involved in diurnal light regulation of plastid gene expression [24,25]. The T-DNA is also inserted in the vicinity (1302 bp upstream) of the coding sequence of a protein with unknown function (At5g24130) (Figure 2A). Publicly available microarray data (eFP browser; [28]; data not shown) suggest that it encodes an almost seed-specifically expressed gene. The direction of the inserted enhancer-trap construct matches with the direction of the *SIG5* gene, which is, like the reporter gene of N9313, leaf expressed and light-responsive [22,29,30]. Based on EST comparison, a 483 bp intron-containing 5'-UTR has been predicted for *SIG5*. Since it is only substantiated by two ESTs (and alternative transcription initiation cannot be excluded), we refer to the translation start site as position +1 for description of the *SIG5* promoter in this manuscript.

The T-DNA insertion site was confirmed by PCR with primers designed to anneal to the genomic DNA flanking the insertion site and to the right border of the T-DNA. The enhancer trap element is inserted 715 upstream of the predicted transcription initiation site of *SIG5* in N9313 and separates the *SIG5* promoter in a proximal part controlling *SIG5* transcription and a distal part regulating *GFP* expression (Figure 2B).

### 2.3. Light Quality Regulation of SIG5 by the Distal and Proximal Upstream Regions

**Figure 3:**
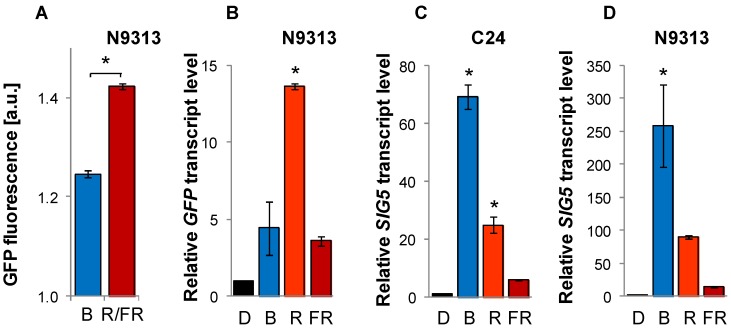
*GFP* and *SIG5* mRNA levels upon illumination with monochromatic light. (A) *GFP* fluorescence in 69–92 10 day old N9313 seedlings illuminated with 10 µmol·photons·m^−2^·s^−1^ blue light or red/far-red light (R/FR); (**B**–**D**) *GFP* and *SIG5* transcript levels in N9313 and C24. Ten day old light grown seedlings were dark adapted for 24 h and then exposed to 100–120 µmol·photons·m^−2^·s^−1^ monochromatic blue, red or far-red light for 24 h. Relative transcript levels were determined by qRT-PCR relative to *ACT2* transcript level and normalized on the transcript levels of dark adapted seedlings prior to the transfer to monochromatic light. Bars represent means (±SEM). * indicates significant differences from the dark adapted samples (Student’s *t*-test, *p* < 0.05).

*SIG5* has previously been described to be blue-light regulated in a light-intensity-dependent manner [29,30,31,32]. To test whether and to which extent blue-light regulation is mediated by the trapped promoter elements, *GFP* fluorescence activity was tested in N9313 plants grown for 10 days in blue light (470 nm, 10 µmol photons m^−2^ s^−1^). The reporter gene activity was only 1.25-fold higher in blue light than in darkness (Figure 3A) demonstrating that it is not sufficient to mediate the strong blue-light induction reported in the literature [30].

In parallel, seedlings were grown under red light (>600 nm). Originally, these plants were thought to function as controls, since it was reported that *SIG5* is not red-light regulated [31]. However, the *GFP* pattern showed that the distal upstream region is sensitive to red light (Figure 3A).

**Figure A1:**
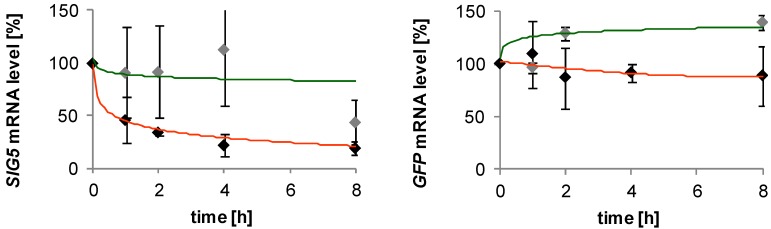
RNA decay analysis. 10 day old seedlings were treated with 200 µM Actinomycin-D in liquid MS-medium for 8 h. The amounts of *SIG5* and *GFP* transcripts were quantified by qRT-PCR and standardized on ACT2.

For further analysis of light quality sensing, *GFP* mRNA levels (resulting from activation of the enhancer trap by the distal upstream region) and the *SIG5* mRNA levels, which are under control of the proximal 715 bp of the upstream region and the *SIG5*-5'-UTR, were compared in the enhancer trap line N9313 and in C24, which is the wildtype background of N9313 and expresses *SIG5* under the full-length promoter. RNA-decay analysis (Figure A1) revealed that during a 24 h treatment 99% of *SIG5* mRNA and 97% of *GFP* mRNA can be expected to depend on *de-novo* synthesis. Here, plants were pre-cultivated for 12 days in 100–120 µmol·photons·m^−2^·s^−1^ white light to minimize germination and early seedling development effects. Afterwards, they were transferred for 24 h into darkness for relaxation and exposed for 24 h to 100–120·µmol·photons·m^−2^·s^−1^ monochromatic blue (471 nm), red (673 nm) or far-red light (745 nm), respectively. Controls were kept in the dark.

Blue light illumination resulted in low *GFP* mRNA levels in N9313 confirming that the distal upstream region is not blue-light sensitive. Consistent with the previous indication for red-light sensitivity of the distal upstream region, stronger *GFP* mRNA accumulation was observed in red-light illuminated N9313 seedlings (Figure 3B). Far-red light resulted in only very low *GFP* expression.

*SIG5* transcript levels, which are under control of the proximal upstream region in N9313, were 260-fold higher in blue light in the enhancer trap line than in darkness showing that the previously reported blue-light sensitivity is mediated within the proximal upstream region. Red light resulted in almost 100-times the dark-level and far-red light gave around 15-times the dark mRNA level of *SIG5* in N9313 (Figure 3D).

In C24 (Figure 3C), the relative red-light and, especially, the far-red-light response were stronger compared to the blue-light reaction than in N9313 (Figure 3D) demonstrating that *SIG5* transcript abundance regulation in C24 combines the regulatory effects of proximal and distal upstream regulation in N9313 (Figure 3B,D).

### 2.4. Arabidopsis SIG5 Transcription Is Red Light Sensitive

**Figure 4:**
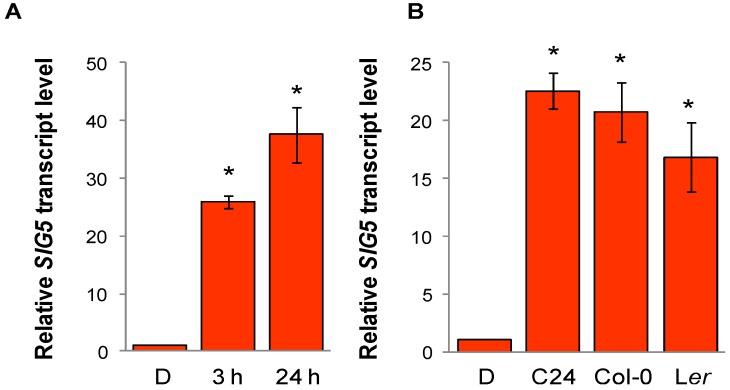
Red-light induction of *SIG5* transcripts. (**A**) Relative *SIG5* transcript levels in rosette leaves of 4 week old N9313 plants. N9313 was grown on soil under continuous white light (20 µmol·photons·m^−2^·s^−1^) for 4 weeks at 22 °C. The plants were dark adapted for 24 h and subsequently exposed to 50 µmol photons m^−2^ s^−1^ monochromatic red light (RL) at 22 °C for 3 h or 24 h; (**B**) *SIG5* transcript level of different Arabidopsis accessions. 10 day old light grown seedlings were dark adapted for 24 h and then exposed to 100–120 µmol·photons·m^−2^·s^−1^ monochromatic red light for 24 h. * indicates significant differences from the dark adapted samples (Student’s *t*-test, *p* < 0.05).

Some of the sigma factors, e.g., *SIG2* and *SIG5*, are strongly induced in far-red light [33], while *SIG5* is the fastest blue-light induced sigma factor. Based on previous analyses, it was more or less excluded that *SIG5* is red-light sensitive [29,30,31,32]. Since most work was done on rosette leaves of 4 week old Arabidopsis plants of the accession *Landsberg erecta* (Ler) and *Columbia-0* (Col-0), while we observed red-light responses in up to 2 week old C24 seedlings, we first adjusted our growth conditions, illumination period and the duration of dark-adaptation and red-light illumination according to [31]. *SIG5* transcript levels were increased in 4 week N9313 plants after 3 h and after 24 h red-light illumination (Figure 4A). In a test for accession specific variation, *SIG5* mRNA levels were also increased in C24, Ler and Col-0 in response to red light (Figure 4B).

### 2.5. In Silico Analysis of the SIG5 Promoter for Putative Light Responsive Elements

In an *in silico* scan of the 2 kb upstream of the CDS of *SIG5* using the PlantCARE [34] and PLACE [35] databases for prediction of plant *cis*-acting regulatory DNA elements, 36 putative light-sensitive motifs were identified (Table 1): An AE-Box (−60) and two GATA-motifs (−71; −163) are located in the intron inserted in the 5'-UTR-region (−56 to −431 relative to the translation insertion site). Thirteen predicted elements map to the proximal upstream region (−483 to −1189). All other motifs were found in the distal upstream region which drives the enhancer trap. Eight predicted motifs cluster between −1428 and −1587. A second hot-spot for light regulated elements was observed between −1687 and −1794 bp upstream of the translation initiation site.

**Table 1:** Light responsive motifs identified in the 2 kb sequence upstream of the CDS of *SIG5* as predicted by PlantCARE and PLACE databases. A graphical overview is provided in Figure A2.

Motif	Sequence ^a^	Position ^b^
AE-box	AGAAACAT (+) AGAAACAT (+)	−60 −1021
GATA motifs	GATA (+) GATA (−) GATA (+) GATA (+)^c^ GATA (−)^c^ GATA (+) GATA (−) GATA (−) ^d^ GATA (−) ^d^ GATA (−) ^d^ GATA (+) ^d^	−71 −163 −441 −841 −871 −914 −989 −1593 −1751 −1944 −1985
Box 4	ATTAAT (−) ATTAAT (−) ATTAAT (−) ATTAAT (−)	−593 −654 −962 −1428
GA motif	AAGGAAGA (−) ^c^	−707
I-box	CACTTATGCT (−) ^c^ aAGATAAGA (−)	−732 −1587
ACE	AAAACGTTTA (−) ^c^ CTAACGTATT (−) ^d^ ACGTGGA (−) ^d^	−763 −1442 −1795
Box I	TTTCAAA (−) TTTCAAA (−) TTTCAAA (+)	−917 −1751 −1901
3-AF1 binding site	AAGAGATATTT (−)	−922
ATCT-motif	AATGTAATCT (+) AATGTAATCT (+) AATGTAATCT (+)	−1205 −1455 −1460
L-box	AAATTAACCAAC (−)	−1426
TCT-motif	TCTTAC (+)	−1541
CATT-motif	GCATTC (+)	−1687
GAG-motif	GGAGATG (−)	−1699
GC-box	CACGTC (+)^d^	−1719
G-box	CACGTG (−)^d^	−1794

^a^ (+) and (−) indicates the sense and complementary strand, respectively. ^b^ positions are relative to the translation start site of *SIG5*. ^c^ *cis*-acting elements within the 196-bp sequence mediating the main blue light response. ^d^ potential HY5 binding sites in the 0.8 kb upstream of the N9313 T-DNA.

### 2.6. Mapping Revealed Locally Restricted Blue-Light Sensitivity and Disperse Red-Light Sensitivity

To map the *SIG5* light sensitive promoter elements, the SALK-collection of T-DNA insertion lines [36] was screened for lines which interrupt the *SIG5* promoter at different positions. Five lines suitable for subsequent analysis were identified: SALK_015625 carries a T-DNA insertion at position −1618 relative to the *SIG5* CDS, SALK_077048 at position −1032, SALK_072457 at −887, SALK_019261 at −691 and SALK_133729 at −515. The T-DNA insertion sites were confirmed by PCR with T-DNA border primers and primers flanking the insertion site. Homozygous lines were isolated from segregating populations by PCR-based genotyping. For the light response tests, 10 day old seedlings grown in white light were dark-adapted for 24 h and treated with monochromatic blue, red and far-red light for 24 h prior to RNA isolation.

**Figure 5:**
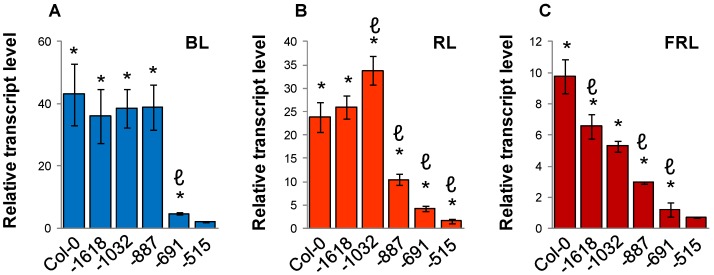
Mapping of the blue-light (BL; **A**), red-light (RL; **B**) and far-red-light (FRL; **C**) sensitive regions of the Arabidopsis *SIG5* promoter by qRT-PCR analysis of *SIG5* transcript levels in Col-0 wild-type and SALK T-DNA insertion lines exposed for 24 h to monochromatic light after 10 days growth in darkness. The numbers give the distances of the T-DNA insertions relative to the *SIG5* CDS. Values were normalized to the *SIG5* transcript level of 24 h dark-adapted seedlings. The data are means of 2–4 biological replicates (±SEM). * indicates significant differences from the dark adapted samples (Student’s *t*-test, * *p* < 0.05). ℓ indicates significant differences from the line with the next longer upstream region (Student’s *t*-test, *p* < 0.05).

In blue-light, the *SIG5* transcript levels were similar in the three T-DNA lines with insertions at nucleotide positions −1618, −1032 and −887 in Col-0 (Figure 5A). Seedlings with T-DNA insertions at position −691 and −515 were strongly impaired in their blue light induction of *SIG5* transcription demonstrating that the 196-bp region between position −887 and position −691 mainly mediates blue-light regulation. Weaker blue-light sensitivity differences were observed between −515 and −691 in the proximal upstream region and upstream of −1618 in the distal region.

When the same lines were compared in red and far-red light, the strongest red-light difference was observed between the −887 and −1032 insertion lines (Figure 5B). Weaker, but still significantly strong positive elements are located between −515 and −691 and between −691 and −887. In the distal upstream region negative red-light regulation was observed if a SALK-line T-DNA was inserted upstream of the position of the enhancer trap insertion in N9313 (−1089), as if the T-DNA insertion destroyed the otherwise inducing element. The overall low far-red-light sensitivity increased gradually with the length of the upstream region (Figure 5C) indicating widely distributed regulation.

### 2.7. Blue- and Red-Light Regulation of the Proximal Upstream Region

**Figure 6:**
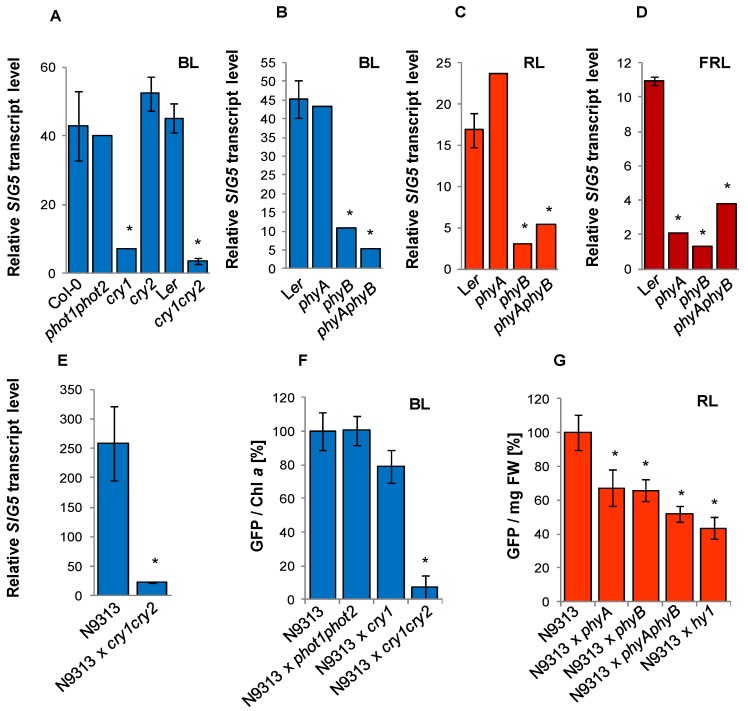
(**A**–**E**) Relative *SIG5* transcript levels in 10 day dark adapted old photoreceptor mutants, their background accessions and N9313 × *cry1cry2* in response to blue (BL), red (RL) and far-red light (FRL) after 24 h exposure to 100–120 µmol·photons·m^−2^·s^−1^ monochromatic light. The transcript levels were determined by qRT-PCR relative to *ACT2* transcript level. The transcript level of dark adapted seedlings was set to 1.0. (F + G) *GFP* fluorescence in N9313 and N9313 crossed with photoreceptor mutants. Relative *GFP* fluorescence of N9313 was set as 100%. Results are mean values of 3–8 measurements (±SEM). * indicates significant differences from the respective control line (Student’s *t*-test, *p* < 0.01).

As a first approach of signal transduction analysis, *SIG5* transcript levels were analyzed in photoreceptor mutant lines (Figure 6) grown next to their respective wild-type for 24 h in monochromatic light after 10 days pre-cultivation in white light and 24 h in darkness. In the *phot1phot2* and the *cry2* mutants, which are deficient in phototropins and cryptochrome 2, respectively [37,38], *SIG5* transcript levels were not significantly different from the respective wildtype lines. In *cry1* and *cry1cry2* mutants [39], it was strongly decreased (Figure 6A) demonstrating that CRY1 is involved in the blue light induction of *SIG5*. *SIG5* transcript levels were also decreased in the homozygous offspring of N9313 × cry1cry2 (Figure 6E) demonstrating that also the weak distal blue-light response is mediated by cryptochromes.

Blue light can alternatively be sensed via activation of the Soret absorption bands of phytochromes [40]. The comparison of *phyA*, *phyB* and the *phyAphyB* double mutants demonstrated that *phyB* mutants are impaired in the blue light induction of *SIG5* transcription, whereas *phyA* mutants are not (Figure 6B).

Comparison of *phyA*, *phyB* and *phyAphyB* mutants in red light showed that the red-light response of the *SIG5*-promoter is mediated by PHYB, but not by PHYA (Figure 6C). Consistent with a general low FR-response, the transcript levels of *SIG5* were low in *phyA*, *phyB* and *phyAphyB* mutants (Figure 6D).

### 2.8. Photoreceptor Control of the Distal SIG5 Upstream Region

**Figure 7:**
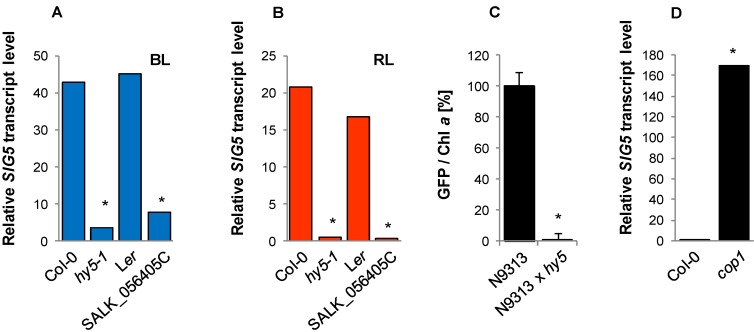
HY5 and COP1 regulation of the *SIG5* promoter in seedlings. (**A** and **B**) Relative *SIG5* transcript levels of old HY5 deficient hy5-1 and SALK_056405C and the respective wild-type in response to blue light or red light. Ten day old seedlings were dark adapted for 24 h and then exposed to 100–120 µmol photons m^−2^ s^−1^ monochromatic blue light or red light for 24 h. Relative transcript level of dark adapted seedlings prior to the transfer to monochromatic light was set to 1.0. (**C**) *GFP* fluorescence in N9313 and N9313 crossed with the HY5 deficient SALK_056405C (mean values of 4–8 samples with 10 seedlings each (±SEM). (**D**) Relative *SIG5* transcript abundances in Col-0 wild-type and in cop1 mutants as determined by qRT-PCR relative to ACT2 values. Light grown seedlings were dark-adapted for 24 h prior to RNA isolation. *SIG5* transcript levels of Col-0 were set to 1.0. * indicates significant differences from the N9313 or Col-0 value (Student’s *t*-test, *p* < 0.01).

For the analysis of the regulatory function of photoreceptors on the distal *SIG5* upstream region, the enhancer trap line N9313 was crossed with *phot1phot2* [37] and with *cry1* and *cry2* [39] mutants. Lines homozygous for the T-DNA and the respective mutant were selected and analyzed for *GFP* expression activity. Due to the severe differences in growth and greening between the mutants and wildtype plants, the reporter gene activities were standardized on chlorophyll-*a* levels after blue-light treatment (Figure 6F) and on fresh weight after red-light treatment (Figure 6G). The *GFP* fluorescence of N9313 x *phot1phot2* mutant plants was similar to that of N9313. The *GFP* values of N9313 × *cry1* were only slightly decreased, while the *GFP* fluorescence of *cry1cry2* double mutants were hardly detectable (Figure 7A), demonstrating that regulation of the distal upstream region depends on the availability of CRY2.

The reporter gene activity was also decreased in crosses of N9313 to *phyA* and *phyB* (Figure 6G). In the *phyAphyB* double mutant it was less than in each single mutant indicating a combined control of the promoter. Besides PHYB, Arabidopsis expresses three more type-II phytochromes. To investigate their overall impact, N9313 was crossed with the *hy1* mutant, which is deficient in phytochromobiline biosynthesis [41]. In response to the *hy1* mutation *GFP* activity was slightly more decreased than by *phyAphyB* (Figure 6G), demonstrating that the distal upstream region of *SIG5* is regulated by the combined action of various phytochromes, with strongest impact of PHYA and PHYB.

### 2.9. Regulation of the Distal SIG5 Upstream Region by HY5 and COP1

**Figure 8:**
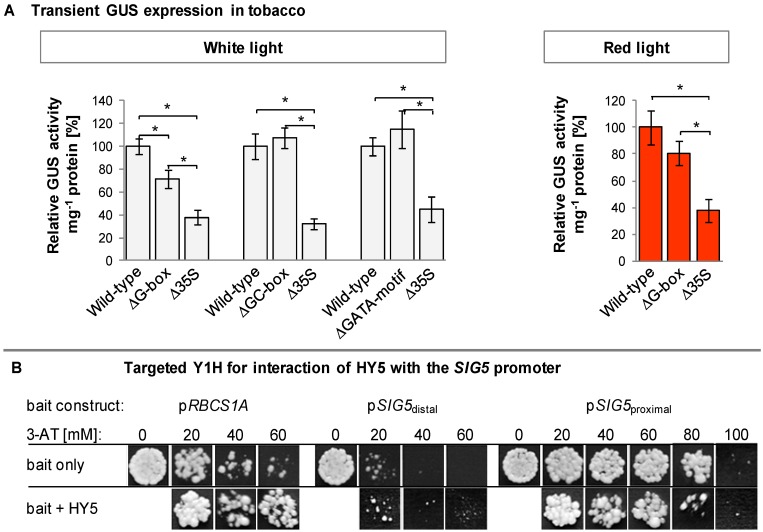
Characterization of potential HY5 binding sites. (**A**) Transient GUS expression as regulated by distal 0.8 kb *SIG5* promoter fragments and variants with differently mutated putative HY5 target elements. Following infiltration, the leaves were illuminated for 5 days with 100–120 µmol photons m^−2^·s^−1^ white light or red light. Statistical significance of difference (Bonferroni’s *post-hoc* test) is indicated as asterisks above bars (*p* < 0.05); (**B**) Yeast-1-hybrid analysis of HY5 interaction with the *RBSC1A* promoter (p*RBCS1A*) and distal and proximal *SIG5* upstream regions (p*SIG5*_distal_, p*SIG5*_proximal_) in Y187. 5 µL of liquid culture with an OD_600_ of 0.01 were analyzed on SD/-H/-T (upper row) or SD/-H/-L/-T (lower row) supplemented with 3-AT. For control, the yeast transformants were cultivated on 3-AT-free SD/-T medium.

The bZIP transcription factor HY5 translates cryptochrome and phytochrome signals into gene expression regulation [42]. Its stability is under control of COP1 [43]. In the two HY5-deficient lines, *hy5-1* and SALK_056405C, *SIG5* transcript abundance was strongly decreased in red and in blue light (Figure 7A,B). qRT-PCR analysis showed a very strong accumulation of *SIG5* transcripts in dark-adapted *cop1-6* seedlings (Figure 8D), confirming that *SIG5* transcription is under control of HY5.

In the homozygous offspring of the cross of N9313 with the *hy5-1* mutant, *GFP* fluorescence was reduced to almost undetectable levels (Figure 7C) demonstrating that HY5 has strong impact on the distal upstream region.

Promoter motif analysis predicted HY5 target sites at the positions −1593 (GATA-motif), −1719 (GC-box) and −1794 (G-box), corresponding to 395, 521 and 596 bp upstream of the N9313 T-DNA insertion site (Table 2). To test whether the predicted motifs are involved in light quality regulation, they were amplified by PCR and fused to a truncated 35S promoter upstream of an open reading frame for *GFP-GUS*. In a PCR-based approach [44] the putative HY5 sites were mutagenized. Young tobacco leaves were transfected with mutagenized and non-mutagenized constructs. Expression activity was quantified from the GUS activity in leaf disks excised from the transfection site. As negative control, the minimal 35S-promoter (without regulatory upstream elements) was fused to *GFP-GUS*. The comparison of mutagenized and non-mutagenized constructs showed white- and red-light sensitivity for the G-box, but not for the other two predicted elements (Figure 8A).

**Table 2:** HY5 binding sites identified in the distal 0.8 kb *SIG5* upstream region and introduced mutations.

*cis*-element	Sequence ^a^	Position ^b^	Introduced mutation ^c^	Reference
G-box	CACGTG (+)	−1794	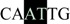	[45]
GC-box	GACGTG (+)	−1719	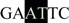	[45]
GATA-motif	GATAAG (+)	−1593	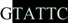	[46]

^a^ (+) and (−) indicates the sense and complementary strand, respectively. ^b^ positions are relative to the translation start site of *SIG5*. ^c^ mutated nucleotides are highlighted in black.

### 2.10. Yeast-1-Hybrid Analysis of HY5 Binding to the SIG5 Promoter

To test for direct binding of HY5 to the *SIG5* promoter, yeast-one-hybrid assays were performed with *SIG5* promoter fragments as baits and HY5 as prey. The promoter of RUBISCO small subunit 1A (pRBCS1A) was used as positive control [43]. HY5 decreased auto-activation of the proximal *SIG5* upstream region in yeast demonstrating binding of the plant transcription factor (Figure 8B). Analysis of the distal *SIG5* upstream region gave no indication for direct HY5 interaction with the distal *SIG5* promoter (Figure 8B mid).

### 2.11. Impact of Photosynthetic Electron Transport on Light Regulation

**Figure 9:**
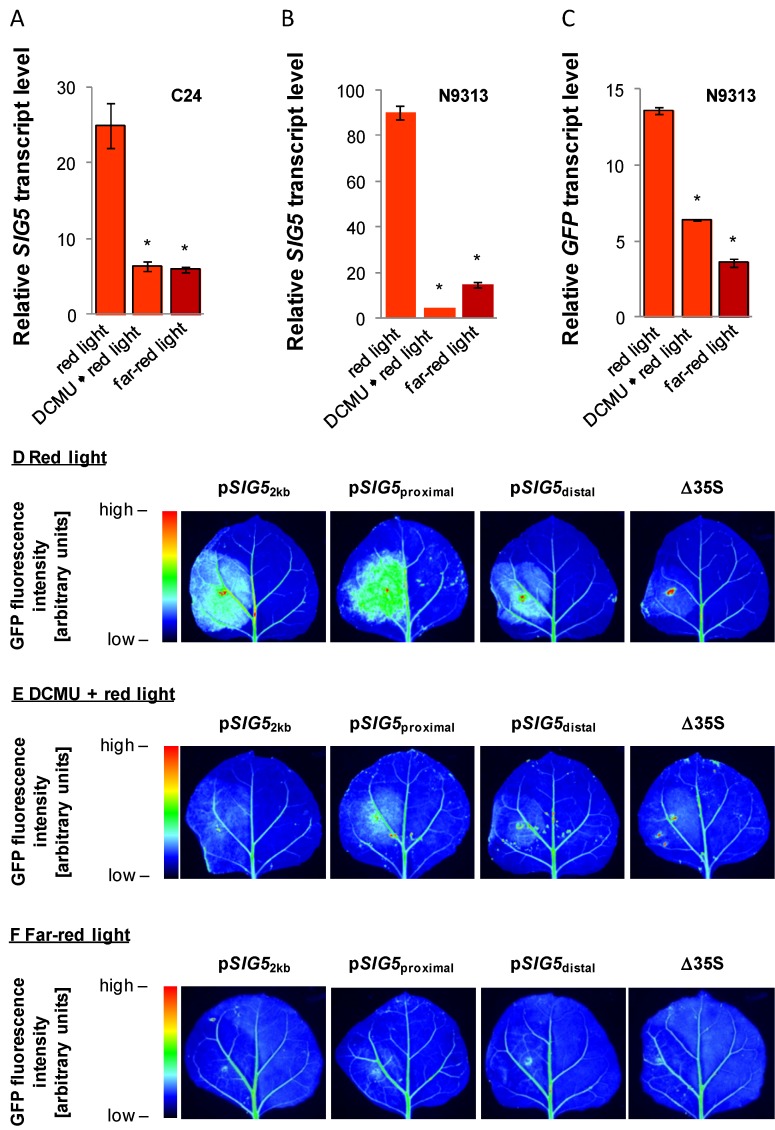
(**A**–**C**) Relative *SIG5* and *GFP* transcript levels in C24 and N9313 in response to red and far-red light and red light 24 h after treatment with 10 µM DCMU and light exposure. The seedlings were pre-cultivated 10 days in 120 µmol photons m^−2^ s^−1^ white light followed by 24 h dark incubation. The transcript levels were standardized on ACT2 transcript levels and normalized to the transcript levels prior to the transfer to monocromatic light. Statistical significance of difference (Student T-Test) is indicated as asterisks above bars (*p* < 0.05); (**D**–**F**) *GFP* expression mediated by the 2 kb full-length *SIG5* promoter, the *SIG5* proximal and *SIG5* distal upstream regions fused to the Δ35S promoter and the Δ35S promoter after 4 days cultivation of the transfected tobacco leaves in monocromatic red and far-red light. In 1/3 of the plants the transfected leaves were sprayed with 1 mM DCMU prior to the exposure to the monochromatic light. The scale of the false color pictures was standardized on the same color scale.

Red light can also drive photosynthesis. Monochromatic red-light is predominantly absorbed by photosystem II [47]. During acclimation, expression of *psaAB*, which encodes the reaction center proteins of photosystem I, decreases and expression of the photosystem-II reaction center protein D1 (encoded by *psbA*) increases in order to optimize photosynthetic efficiency [48]. *SIG5* regulates transcription of the photoreaction center proteins D1 and D2 (encoded by *psbA* and *psbD*) [24,32]. Here, *SIG5* expression was shown to be light-intensity dependent (Figure 1) and red-light regulated (Figure 9).

To test the importance of photosynthetic electron transfer on *SIG5* expression, half of the 8 day old seedlings grown in white light were sprayed with 10 µM DCMU prior to shifting them for 24 h to red light. DCMU blocks the Q_B_-site [49]. Electrons cannot be transferred from photosystem II to the plastoquinone pool. Therefore, DCMU should simulate over-excitation of photosystem II and function antagonistically to non-saturating doses of red light.

In C24, DCMU application decreased red-light dependent induction of the *SIG5* promoter (Figure 9A). In N9313 the *SIG5* transcript levels were hardly detectable after DCMU treatment (Figure 9B). The results demonstrated that regulation of the proximal upstream region depends strongly on photosynthetic electron transport. Red-light regulation of the distal upstream region is only partly dependent on electron transport activity as indicated from a weaker decrease in response to DCMU (Figure 9C).

To test how strong the regulation by photosynthetic electron transport is manifested in the promoter, red-light- and DCMU-responses were analyzed in young tobacco leaves which were transfected with constructs expressing *GFP* under the control of the *SIG5* full length promoter (Figure 9D,E), the distal or proximal upstream regions (fused to the Δ35S minimal promoter), or the Δ35S minimal promoter. Similar as in seedlings, DCMU antagonized the red-light dependent induction of the full-length, the distal and the proximal *SIG5* upstream regions.

**Figure 10:**
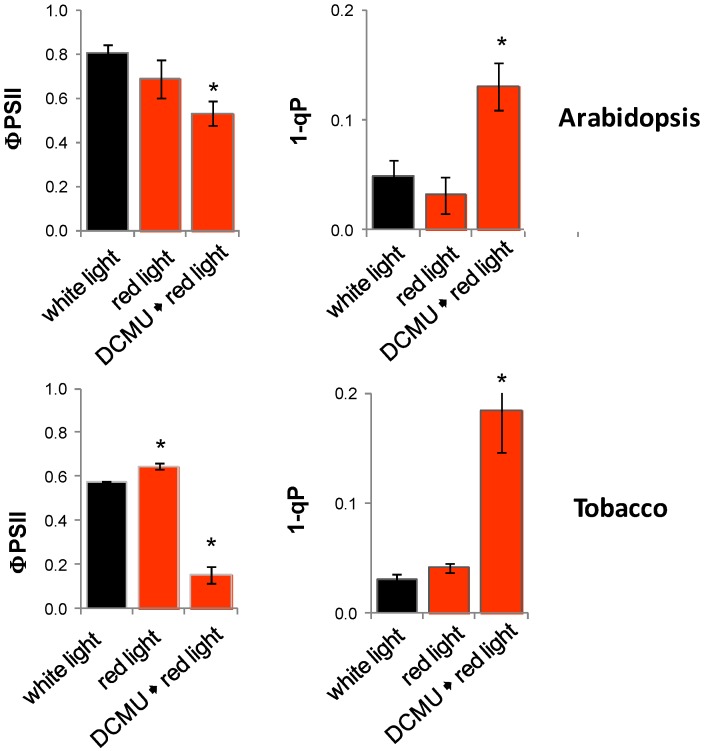
Photosynthetic performance upon light and DCMU treatment. (**A**) Quantum yield of photosystem II following dark adaptation; (**B**) Redox state of the plastoquinone pool. Arabidopsis was grown for 10 days in 100–120 µmol photons m^−2^ s^−1^ white light. Following 24 h dark relaxation, they were transferred to red or white light for 24 h. Half of the plant transferred to red light were treated with DCMU prior to the light shift. DCMU-treated and non-treated tobacco leaves were incubated for 4 days in 100–120 µmol photons m^−2^ s^−1^ white light or red light. Statistical significance of difference (Student’s *t*-test) is indicated as asterisks above bars (*p* < 0.05).

In non-transfected plant material, the redox state of the plastoquinone pool (1-qP) and the quantum yield of photosystem II (F_V_/F_M_) were determined (Figure 10). The redox state of the plastoquinone pool was highly oxidized in red light in the presence and absence of DCMU. In red light the quantum yield of photosystem II was slightly higher than in white light in tobacco and in the range of white light treated plants in Arabidopsis in absences of DCMU and decreased after DCMU treatment reflecting PSII damage.

### 2.12. Sugar Effect on SIG5 Transcription

Intensity dependent light responsiveness can be mediated by photosynthate signatures. *Vice* *versa*, high levels of carbohydrates can indicate high photosynthetic activity. As part of the feed-back mechanisms, they inhibit the activity of the Calvin-Benson-Cycle and, therefore, the main sink for electrons transported in the photosynthetic light reaction. The plastoquinone pool is more reduced and the quantum yield on photosystem II decreased [50].

**Figure 11:**
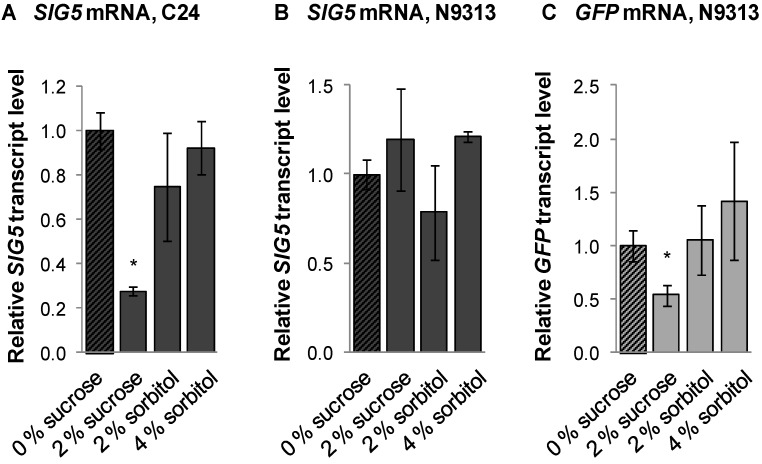
Relative *SIG5* and *GFP* transcript levels in C24 and N9313 in response to sucrose and sorbitol. The transcript levels were standardized on ACT2 and normalized on the transcript levels determined on 0% sucrose. Statistical significance of difference (Student’s *t*-test) is indicated as asterisks above bars (*p* < 0.05).

The impact of carbohydrates on the light-regulated *SIG5* promoter was tested by application of sucrose. The osmotic side effects of sucrose were simulated by sorbitol. Forty eight hours after application of sucrose or sorbitol to 10 day old light-grown seedlings, the *SIG5* mRNA and *GFP* mRNA levels were quantified in C24 and N9313. Sucrose resulted in decreased *GFP*-levels in N9313. *SIG5* transcript amounts were decreased in C24, but not in N9313 (Figure 11) demonstrating that only the distal upstream region is sucrose-responsive. Sorbitol did not alter *SIG5* expression significantly.

## 3. Experimental

### 3.1. Plant Material and Growth Conditions

Seeds of all SALK-lines, Arabidopsis wildtype accessions (C24, Col-0 and L*er*), the *GAL4 GFP* enhancer trap population, and the mutants *cry2*, *hy1-1* and *hy5-1* were obtained from the Nottingham Arabidopsis Stock Centre (NASC, Nottingham, UK). Other mutants were kindly provided by Prof. A. Batschauer (Phillips-Universität, Marburg, Germany).

For sterile growth, Arabidopsis seeds were surface sterilized for 1 min in 80% (v/v) ethanol followed by 8 min in 20% (v/v) household bleach. After five washing steps in sterile water, they were transferred on sterile MS medium (pH 5.7) (Duchefa, The Netherlands) supplemented with 2.5 g L^−1^ Phytagel (Sigma, Taufkirchen, Germany) and 0.5% (w/v) sucrose. Unless otherwise described, experiments were performed with seedlings grown under short day conditions (10 h light, 22 °C/14 h dark, 18 °C) at 120 µmol photons m^−2^ s^−1^ white light (Philips F17TS/TL741 ALTO) after two days of stratification. Alternative light regimes were performed in FloraLED systems (CLF PlantClimatics, Wertingen, Germany) or growth cabinets in blue light (471 nm), red light (673 nm) and far-red light (745 nm).

For sucrose and sorbitol effector studies, 12 day old seedlings grown on sucrose-free media in white light (Philips F17TS/TL741 ALTO; 100 µmol photons m^−2^ s^−1^) were transferred to media supplemented with 2% sucrose and 2% and 4% sorbitol, respectively. The plants were harvested after 24 illumination with white light.

*Nicotiana benthamiana* plants were grown on soil for four to five weeks in a day/night regime of 16 h light and 8 h darkness. Light intenstity and light quality studies were performed in FloraLED systems or growth cabinets in blue light (471 nm), red light (673 nm), far-red light (745 nm) or white light (Philips F17TS/TL741 ALTO, 17 watt).

### 3.2. Extraction of Genomic DNA, TAIL-PCR and Sequence Analysis

For small scale isolation of genomic DNA, plant material was homogenized in 200 µL 50 mM Tris/HCl pH 8.0, 25 mM EDTA, 250 mM NaCl and 0.5% (w/v) SDS. After extraction with 200 µL phenol-chloroform-isoamyl alcohol (25:24:1), the samples were centrifuged at 20,000 ×g for 5 min at room temperature. The DNA was precipitated for 1 h at −20 °C after transfer of the upper phase to 200 µL isopropanol. Following 15 min centrifugation at 20,000 ×g, the precipitated DNA was resuspended in 100 µL water.

TAIL-PCR was performed with 2 µL DNA solution per 20 µL total volume as described in [27] with the primers listed in Table 3. PCR products were cloned into pJET1.2/blunt (Fermentas/Thermo-Fischer, St. Leon-Rot, Germany). Sanger sequencing was performed by GATC (Konstanz, Germany). Database searches were performed using the BLAST program on the NCBI server [51]. The insertion sites were confirmed by PCR, designed to anneal the genomic DNA flanking the mapped insertion sites and T-DNA primer TAIL-TR3.2.

### 3.3. Southern Blot Analysis

DNA was extracted from plant material using the DNeasy Plant Mini Kit (Qiagen, Hilden, Germany). Three µg of genomic DNA was digested with *Bgl*II (Fermentas) and purified by sodium acetate precipitation prior to separation on 1% (w/v) TAE agarose gels and capillary transfer onto Hybond^TM^-N membranes (GE-Healthcare, Freiburg, Germany). A 504 bp digoxigenin (DIG) labeled *GAL4* probe was amplified from genomic DNA of N9313 with the DIG Probe Synthesis Kit (Roche, Germany) by PCR using primers mPPR1-5 (5'-CGGCAAGCTTGGATCCAACAATG-3') and mPPR1-3 (5'-CCCGGAGCTCGTCCCCCAGGCTG-3'). After crosslinking of the DNA to the membrane by UV-light, the blot was hybridized under high stringency conditions using DIG Easy Hyb Granules (Roche, Mannheim, Germany). Following two washing steps at 68 °C in 75 mM NaCl, 7.5 mM sodium citrate (pH 7.0) with 0.1% (w/v) SDS and two in 15 mM NaCl, 1.5 mM sodium citrate (pH 7.0) with 0.1% (w/v) SDS, the bound probes were detected in a Luminescent Image Analyzer LAS-4000 (GE-Healthcare) using the CDP-Star, ready to use Kit (Roche).

### 3.4. Identification and Isolation of Homozygous T-DNA Insertion Lines by PCR

T-DNA insertion lines were tested for the T-DNA insertion site and homozygosity by PCR with T-DNA border primer LBb1.3 (5'-ATTTTGCCGATTTCGGAAC-3') and specific primers (Table 3) binding upstream and downstream of the predicted T-DNA insertion site. For confirmation, the PCRs were repeated with 10 seedlings of the progeny.

**Table 3:** Primers and annealing temperatures used for PCR.

PCR reaction/ product	T_A_ [°C]	Forward primer/reverse primer (5'→3')
TAIL-PCR, T-DNA primers	73.1	CACTTGGCGCACTTCGGCTTC
	67.5	AGCTTCTTGAGGCGGCAGA
	63.3	GGAGCTTCATTGTTGGATCC
TAIL-PCR, AD primers	46.0	NTCGA(G/C)T(A/T)T(G/C)G(A/T)GTT
	46.8	NGTCGA(G/C)(A/T)GANA(A/T)GAA
	34.8	(A/T)GTGNAG(A/T)ANCANAGA
Verification of N9266 T-DNA	58	CGTATCACGCGGCGC
Verification of N9313 T-DNA	54	CTCCGACTCTTGCGATAT
qRT-PCR, *ACT2*	60	TCTTCCGCTCTTTCTTTCCAAGC
		ACCATTGTCACACACGATTGGTTG
qRT-PCR, *SIG5*	60	TGGAGCTAATAACAGCAGACAGC
		TCGGCTTCAATGAATCGAGCAC
qRT-PCR, *GFP*	60	CCATTACCTGTCCACACAATC
		GTTCATCCATGCCATGTG
qRT-PCR, *HY5*	60	AGAACAAGCGGCTGAAGAGGTTG
		TCCTCTCTCTTGCTTGCTGAGCTG
Construction of p*SIG5*_wildtype_	45	ACTAGTTTTTTCTGCAGGTAACTCCGACTCTTGCG
		GCTTGAGAGATTACATTATT
Construction of Δ35S	62	TTCGCAAGACCCTTCCTCTATATAAGG
		GGGTACCGGTCGCCACC
Analysis of SALK_015625	50	CAATCATGGTTTAATTCGT ^a^
		GATCCACAACCACAAGCC
Analysis of SALK_077048	45	GTTATTGATCTGTACCTAGC ^a^
and 072457		AAATACGATAGATGTGTTG
Analysis of SALK_019261	45	ATCACAATCTTAAGGCTCAAAA
		AAATACGATAGATGTGTTG ^a^
Analysis of SALK_133729	45	ATCACAATCTTAAGGCTCAAAA ^a^
		AAATACGATAGATGTGTTG
Identification of *cry2-1* allele	58	CAGTTTTATCCTGGAAGAGCCTC
		CTTCTCCTTTACGGTATGGTCC
Identification of *hy1-1* allele	56	GGAATTAGCAGAGAAGGATCC
		TATCCGCTCTGCCACCTG
Identification of *phyA-201* allele	55	CCTTAAATGAAGTGTTGACTGC
		GCAAGATGCACAGAACG
Identification of *phyB-5* allele	55	GTTGTGGAGTGGTTGCTTG
		CATAGCCGCCTCAGATTC
Identification of *phot1-5* allele	58	CCACTTGCAACCTATGCG
		CTCTTTCACTGCGGTTTCTTC
Identification of *phot2-1* allele	54	CTCTGCCTCACAATAAGGAG
		CTGCCAGTATCACCAGAGC
Identification of SALK_056405C	58	GCGGTAGCCAGAGTAATCTATTCC
		TCCTCTCTCTTGCTTGCTGAGCTG
		ATTTTGCCGATTTCGGAAC ^b^
Construction of p*SIG5*_ΔGATA_-1a	60	GGTGGCGACCGGTACC
		CATCTTTTACTGAATACTTTGAGTTATTTGCACATATAG
Construction of p*SIG5*_ΔGATA_-1b	55	CTATATGTGCAAATAACTCAAAGTATTCAGTAAAAGATG
		GTAACTCCGACTCTTGCGAT
Construction of p*SIG5*_ΔG_-1a	60	GGTGGCGACCGGTACC
		CTGAGAAGACCATCCAATTGTATAATTCCTGATC
Construction of p*SIG5*_ΔG_-1b	55	GATCAGGAATTATACAATTGGATGGTCTTCTCAG
		GTAACTCCGACTCTTGCGAT
Construction of p*SIG5*_ΔGC_-1a	60	GGTGGCGACCGGTACC
		CTATAAATTGGCCAATTCGTCTCTCTCTC
Construction of p*SIG5*_ΔGC_-1b	55	GAGAGAGAGACGAATTGGCCAATTTATAG
		GTAACTCCGACTCTTGCGAT
Construction of p*SIG5* fragments-2	63	GGTGGCGACCGGTACC
		GTAACTCCGACTCTTGCGAT
Y1H-construction of p*RBCS1A*	55	TTTTTGAGCTCGATTTTGAGTGTGGATATGTGT
		TTTTTGAATTCCCAGGCAAGTAAAATGAGCAAG
Y1H-construction of HY5 CDS		TTTTTGGATCC**TA**CAGGAACAAGCGACTAGCTC ^c^
		TTTTTCTCGAGTCAAAGGCTTGCATCAGC
Y1H-construction of p*SIG5_distal_*	57	TTTTTGAGCTCCACAATCTTAAGGCTCAAAAATTG
		TTTTTGGGCCCTCGGATGCTTTACATGGTG
Y1H-construction of p*SIG5_proximal_*	60	TTTTTGAATTCGTAACTCCGACTCTTGCG
		TTTTTGAGCTCGCTTGAGAGATTACATTATT

^a^ Leading to amplification of a T-DNA specific PCR product when combined with primer LBb1.3 annealing with the left border of the SALK T-DNA. ^b^ LBb1.3, annealing with the left border of the SALK T-DNA. LBb1.3 leads to a PCR product in SALK_056405C if combined with the reverse primer annealing the HY5 coding sequence. ^c^ two nucleotides added to HY5 CDS are marked in bold.

### 3.5. Determination of Reporter Gene Activity

*GFP* activity screens were performed in 96-well microtiter plates on 100 µL MS medium per well supplemented with 0.5% (w/v) sucrose in a Fluoroskan Ascent FL fluorometer (Thermo Fisher Scientific, St. Leon-Rot, Germany) in the top-reader modus with 500 ms integration time, 485 nm excitation at 527 nm. Each well was covered with nine measurement points to scan all plants equally. Background fluorescence was subtracted from the means by analyzing the autofluorescence at 527 nm of parallel grown C24 seedlings. Normalization was performed based on area determination using the ImageJ software package [52].

In crosses of N9313 with the photoreceptor mutants, the *GFP* activity was quantified in triplicates from 100 µL extracts because of the high variability in seedling morphology. Plant material (10–20 mg) was homogenized in 500 µL sodium phosphate, pH 7.0. After 2 min centrifugation at 20,000 ×g *GFP* fluorescence was quantified in a Mithras LB 940 top reader fluorometer (Berthold Technologies GmbH & Co. KG, Bad Wildbad, Germany) with 100 ms counting time (excitation filter 460/10 and emission filter F510). Chlorophyll contents were determined according to [53].

*GUS* expression was quantified in 15–30 mg leaf material and standardized on protein levels as described in [54].

### 3.6. Transcript Abundance Analysis

RNA was isolated from 10–15 frozen seedlings in Precellys^®^ 24 (Peqlab, Erlangen, Germany) using the RNeasy Plant Mini Kit. The purity of the RNA was determined spectrophotometrically from the A_260_/A_280_ ratio. cDNA was synthesized with the High-Capacity cDNA Reverse Transcription Kit (Applied Biosystems, Darmstadt, Germany). Primers for qRT-PCR were designed by the QuantPrime software [55,56] (Table 3). The primer specificity was checked by assaying that the melting curves display only a single peak for each PCR product of interest, and by gel electrophoresis. Real-time amplification was performed according to the MIQE standards [57] using the Brilliant II SYBR^®^ Green Master Mix (Agilent Technologies, Böblingen, Germany) in a Stratagene MX3005P Cycler (Agilent Technologies, Böblingen, Germany). No-template-controls were performed for each gene. Each biological replicate represents an independent RNA isolation. Levels of each transcript relative to the constitutively expressed *ACT2* control gene [58] were quantified as described by Pfaffl [59].

### 3.7. mRNA Decay Analysis

Transcription was inhibited by transferring 10 day old N9313 seedlings to liquid MS medium containing 200 µM Actinomycin D (Act D) (Sigma-Aldrich, Taufkirchen, Germany) at 120 µmol photons m^−2^ s^−1^. Control plants were incubated in MS medium. The mRNA half-life was quantified for *SIG5* and *GFP* transcripts from the C_t_ values determined by *ACT2*-standardized qRT-PCR in samples treated for 1, 2, 4 and 8 h. Since mRNA decay generally obeys first-order kinetics [60,61], an exponential regression model (A = 1e^−kt^) was applied for determination of the decay coefficient (k_decay_). The mRNA half-life was then calculated using the following equation: t½ = ln(2)/k_decay_.

### 3.8. Reporter Gene Construct Design, Site-Directed Mutagenesis and Tobacco Transfection

*SIG5* promoter fragments were amplified from genomic DNA of the accession C24 by PCR with specific primers containing *Pst*I and a *Bcu*I restriction sites (Table 3) and cloned into the TA cloning site of the pCR8/GW/TOPO vector (Invitrogen, Darmstadt, Germany). The truncated (–48) 35S minimal promoter from cauliflower mosaic virus (CaMV) which was amplified by PCR from T-DNA of the enhancer trap line and cloned into the restriction sites. The promoter fragments were transferred into the binary vector pHGWFS7.0 [62] with LR Clonase II enzyme mix (Invitrogen, Darmstadt, Germany). *Agrobacterium tumefaciens* strain GV3101 [63] was transformed using the freeze-thaw method [64].

For transfection of tobacco leaves [65], transformed *Agrobacteria* were cultivated in 10 mL YEB medium at 28 °C and 180 rpm to OD_600_ 0.5. For co-infiltrations, GV3101(pMP90) and the respective *SIG5*-promoter strain were mixed in a 2:3-ratio. Cells were collected by 8 min centrifugation at 3000 ×*g* at room temperature and resuspended in 100 mM MES pH 5.6 plus 10 mM CaCl_2_ supplemented with 150 µM acetosyringone. After 2 h incubation, the agrobacteria suspensions were infiltrated into tobacco leaves according to [66]. Site-directed mutagenesis was performed according to [44].

### 3.9. In Silico Analysis of Promoter Sequences

Promoter motif searches were performed *in silico* using PLACE [35,67] and PlantCARE [34,68].

### 3.10. Yeast One-Hybrid Analysis

*SIG5* and *RBCS1A* promoter fragments and *HY5* cDNA were generated by PCR using genomic DNA of the ecotype C24 as template. The *HY5* cDNA was cloned into the *Bam*HI*/Xho*I site of pACT2 (Clontech, Montain View, CA, USA). *SIG5* promoter fragments and a 196-bp fragment of the Arabidopsis *RBCS1A* promoter were cloned into the *Sac*I/*Eco*RI site of pHIS2 (Clontech, Mountain View, CA, USA) using the LigaFast Rapid DNA Ligation System (Promega, Fitchburg, Madison, WI, USA). The *Saccharomyces cerevisiae* strain Y187 (*MATα*, *ura3-52*, *his3-200*, *ade2-101*, *trp1-901*, *leu2-3*, *112*, *gal4Δ*, *met-*, *gal80Δ*, *URA3:GAL1_UAS_GAL1_TATA_-lacZ*, *MEL1*, with reporter genes *HIS3* and *lacZ*) was transformed with lithium acetate (LiAc) [69]. Transformants were selected on minimal synthetic dropout (SD) medium (Clontech, Montain View, CA, USA) lacking the amino acids corresponding to the autotrophy markers of the plasmids. For yeast colony-PCR, a single colony was resuspended in 50 µL water supplemented with 60 U mL^−1^ lyticase and incubated at 30 °C for 30 min. After cell lysis at 95 °C (10 min) and 2 min centrifugation at 20,000 ×*g*, 2 µL of the supernatant was used for PCR in a total volume of 20 µL.

For the interaction tests, 5 µL of overnight cultures of double-transformed yeast stains (adjusted to OD_600_ = 0.01) were dropped onto SD/-His/-Trp plates containing 10–100 µM 3-amino-1,2,4-triazol (3-AT) to increase the stringency. Plates were incubated 2–3 days at 30 °C.

### 3.11. Crossing Arabidopsis thaliana Plants and Mutant Selection

Arabidopsis lines were cross-pollinated. From the progenies, homozygous *cry1*, *phyA* or *phyB* mutants were selected according to their hypocotyl length in blue, red or white light, respectively [18]. Plants carrying the homozygous *phot1* allele were identified by measuring the phototropic response [70]. The presence of the *GFP*-enhancer element was tested by PCR.

### 3.12. Fluorescence Microscopy

Tissue-specific *GFP* expression was analyzed by confocal laser scanning microscopy using a Zeiss LSM 510 Meta with a multiline argon ion laser (Carl Zeiss, Jena, Germany). Cells were examined with a 40X Zeiss oil-immersion objective (1.3 numerical aperture). *GFP* was excited at 488 nm and the emission was recorded through the meta-channel at 497–550 nm. Fluorescence images were analyzed with LSM Image Browser software [71].

### 3.13. Chlorophyll-a Fluorescence Analysis

The quantum yield of photosystem II and the redox state of the plastoquinone pool were determined with a Dual-PAM-100 (Heinz Walz, Effeltrich, Germany) at room temperature after 20 min dark incubation with saturating light flashes of 6000 µmol photons m^−2^ s^−1^ as F_V_/F_M_ [72]. The redox state of the plastoquinone pool was calculated from the steady state photochemical quench (qP) in 100 µmol photons m^−2^ s^−1^ red light (600 nm) as (1-qP) with light flashes of 6000 µmol photons m^−2^ s^−1^.

## 4. Discussion

### 4.1. Transcriptional Regulation of SIG5 Promoter

*SIG5* is one of six sigma factors of Arabidopsis [73,74] controlling plastid gene expression [75]. It activates *psbD* and *psbA* transcription upon dark-light shifts and controls diurnally chloroplast gene expression in a light-dependent manner [24,25,32]. Whereas the structure of the *psbD* promoter was a subject of intensive investigation [76,77], the structure of the *SIG5* promoter has been barely investigated. From transcript abundance analysis [24,29,30] and GUS-reporter gene assays [32], *SIG5* transcription was shown to be strongly blue-light responsive and stress-sensitive. Here, we showed that it is also red-light responsive in a photoreceptor- and photosynthesis-dependent manner (Figure 6, Figure 7 and Figure 9).

In blue light, *SIG5* is the fastest transcriptionally activated sigma factor [30]. *SIG5* binds to the −948 upstream region of the *psbD* promoter, which drives the *psbD-psbC-psbZ* operon encoding core-proteins of photosystem II, in response to blue and white light and various stress conditions [32,76]. *SIG5* is hardly expressed in far-red light (Figure 3C and [33]) and, therefore, can be assumed to be less important than other sigma factors, especially *SIG2* and *SIG6*, in shade avoidance reactions, such as hypocotyl elongation, and activation of greening upon germination [78,79]. As shown by promoter-GUS analysis [32], *SIG5* transcription activity quickly decreases three and four days after onset of germination and becomes activated again in rosette leaves. *SIG5* transcript abundance increases in response to high light, NaCl, mannitol and cold in rosette leaves [32]. Analysis of *SIG5*-deficient T-DNA insertion lines demonstrated that *SIG5* is essential for maintaining photosynthetic activity upon stress [32].

In contrast to the blue/far-red type of sigma factors, e.g., *SIG2* and *SIG6*, *SIG5* is induced by red and blue light (Figure 3 and Figure 5). The regulatory elements are spread widely over the proximal and distal upstream region. Comparison of distal and proximal regulatory activities (Figure 3) and stepwise deleted promoters (Figure 5) show complex combinational regulation. Most of the red-light induction, part of the photosynthetic control and the sugar regulation take place in the distal upstream region (Figure 3, Figure 9 and Figure 11), while the proximal upstream region mediates the main blue-light sensitivity (Figure 5).

### 4.2. Blue-light Regulation

The 196 bp blue-light responsive region, located 208–404 bp upstream of the predicted transcription initiation site (Figure 5A), is under control by CRY1 and PHYB (Figure 6A,B). CRY2 and the phototropins PHOT1 and PHOT2 are of negligible importance (Figure 6A).

The CRY2-mediated blue-light responsive enhancers of the distal upstream region (Figure 3A,B and Figure 5A) may support CRY1 regulation of the distal upstream region (Figure 7A) under low light, such as upon shading or during germination. In the rosette stage, Arabidopsis leaves are frequently exposed to high light intensities. Under these conditions, CRY2 can be expected to be light inactivated [80,81].

### 4.3. Red-light Sensitivity

*SIG5* transcription and transcript abundance were regulated by various phytochromes via widely dispersed red-light sensitive promoter elements (Figure 5 and Figure 6). The region −887 to −1032 in the proximal upstream region showed preference toward PHYB-mediated regulation (Figure 6B), which is also the most abundant phytochrome in light-grown *Arabidopsis* [82].

In blue-light around 78% and in red-light 59% of the *SIG5* transcripts depended on PHYB after 24 h (Figure 6B,C). In a similar experiment, in which light-grown Arabidopsis plants were transferred back to light after 30 h dark treatment, *SIG5* transcript levels were wild-type-like in *phyA* and *phyB* mutants after 1.5 h of illumination [32]. Differences can be observed after 6 h. At this time, the overall *SIG5* expression was low in the analysis [32]. The comfortable access to a wide range of transcript abundance studies from various labs via tools like the eFP browser [28] showed strong diurnal regulation of *SIG5* expression. Therefore, we compared transcript levels exactly after 24 h (except Figure 4A; 3 h data) and harvested plant material at the time of highest transcript abundance 2–4 h after onset of light. This approach demonstrated that *SIG5* expression is also strongly red-light sensitive. Light signals are transmitted mainly via CRY1, PHYA and PHYB in a HY5 and COP1 dependent manner. Consistent with ChIP-based prediction of HY5-binding sites in the *SIG5* promoter, [83] and previously shown HY5-dependent transcript abundance regulation [32], HY5 binds to the proximal upstream region (Figure 8B) and a HY5-binding motif, a G-box, was shown to transmit white- and red-light signals (Figure 8A).

### 4.4. Antero- and Retrograde Signaling Connecting Chloroplast and Nuclear Gene Expression

*SIG5* expression is promoted in red- and blue-light (Figure 3) and in a light intensity dependent manner (Figure 1). Depending on *SIG5* regulation, chloroplast gene expression and photoprotection are regulated [24,32] indicating that its main function of *SIG5* is anterograde light acclimation, such as compensation of photosynthetic imbalances due to suboptimal photoreaction center innervation.

In addition to the anterograde function [32], *SIG5* is regulated by retrograde signals (Figure 9): Red light preferentially activates photosystem II [84]. Blocking the Q_B_-site of photosystem II with DCMU [49], decreased activation of *SIG5* expression in red light (Figure 9). In red light and after DCMU treatment, the plastoquinone pool was oxidized as determined by chlorophyll-a fluorescence (1-qP; Figure 10). This result excludes the possibility of regulation by the redox state of the plastoquinone pool and showed that full *SIG5* expression depends on photosynthetic electron transfer from photosystem II to the plastoquinone-pool.

*psbA* and *psaAB* expression respond to light-quality changes in order to adjust photoreaction center I and II availability to the relative innervation of the photosystems [84]. Transcription of *psaAB* is under control of the redox state of the plastoquinone pool, but not *psbA* [85]. *SIG5* controls expression of *psbD* and, less specifically, *psbA* [32]. It is the anterograde control factor [32] which can adjust plastid gene expression to light signals due to its complex light quality sensitivity (Figure 1, Figure 3 and Figure 5).

Excess carbohydrates inhibit photosynthetic electron transport [50,86]. Like DCMU, sucrose suppresses *SIG5* expression (Figure 11). If *SIG5* would be regulated depending on the redox state of the plastoquinone pool, sucrose application should have resulted in a higher reduction status. The sucrose data give additional evidence for independence from plastoquinone redox regulation, which controls state transition [87], expression of photosystem I core proteins [84] and STN7-dependent regulation of nuclear gene expression [88]. Regulation of *SIG5* expression by red light and DCMU (Figure 9 and Figure 11) demonstrates that transcription of the nuclear encoded *SIG5* gene responds to photosynthetic signals.

**Figure 12:**
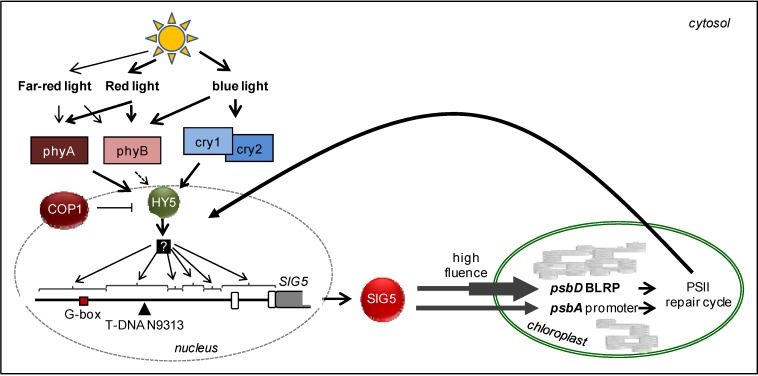
*SIG5* expression control in the light-signaling pathway regulating the expression of the chloroplast genes *psbD* and *psbA* for the photoreaction center II core proteins D1 and D2 in response to light intensity and quality. Light signals regulate *SIG5* expression by photoreceptors in a HY5- and COP1-controlled manner via various promoter elements. *SIG5* is post-translationally targeted to chloroplasts, where the sigma factor is involved in tuning expression of *psbD* and *psbA*. Photoreaction center expression is involved in the PSII repair cycle. Retrograde signals transmit information correlating with the photosystem II excitation status and damage, respectively, to the nucleus to adjust the intensity of *SIG5* transcription.

The antagonism of the sucrose effect to the red-light effect excludes carbohydrates as direct signaling molecules in chloroplast-to-nucleus transmission of the photosynthetic electron transport signal. Insensitivity to similar concentrations of sorbitol (Figure 11B,C) and induction by higher concentrations of sorbitol (Figure 11C) and mannitol [32] excludes osmotic signaling. The comparison of the red-light and DCMU response (Figure 3 and Figure 9) and the general stress-induction reported before [32], which decrease photosystem II activity [89], let assume that the signal emerges at PSII in response to high excitation pressure. A link between generation of signaling by D1 damage or the D1 repair cycle, demonstrates that *SIG5* controls a perfect regulatory circuitry: In chloroplasts, D1 damage reflects over-excitation of photosystem II [90]. In response to retrograde signals, *SIG5* drives expression of D1 and its closest protein partners D2, *PsbC* and *PsbZ* [24,32] and stabilizes photosystem II activity (Figure 12).

## 5. Conclusions

*SIG5* expression is regulated by multiple light-responsive elements spread over the 2 kb upstream region (Figure 5). Signal transduction is controlled by cryptochromes, especially CRY1, and phytochromes (Figure 6) in a HY5 and COP1-dependent manner (Figure 7) and by photosynthetic signals (Figure 9). We conclude that the chloroplast sigma factor *SIG5* is a retrograde and light controlled regulator of chloroplast function. It combines intrinsic and extrinisic information important in adjusting nuclear and plastid gene expression upon light acclimation processes and links the signaling and regulation potential of the eukaryotic-type extra-plastidic gene expression system with the chloroplast regulatory system.

## Figures and Tables

**Figure A2:**
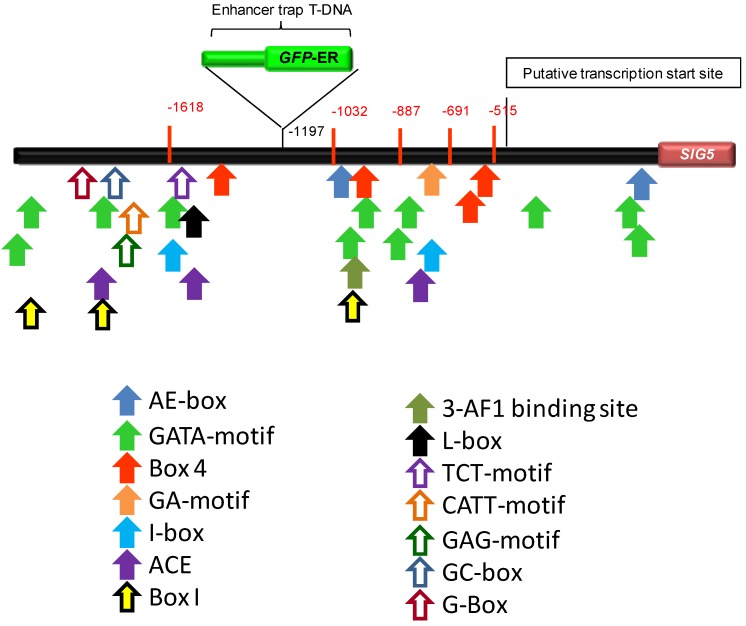
Graphical overview of the distribution of motifs in the *SIG5* upstream region. Information on the precise positions of the motifs is listed in Table 1.

## Abbreviations

3-AT: 3-amino-1,2,4-triazol
Act D: actinomycin D
ANOVA: analysis of variance
BL: blue light
BLRP: blue light responsive promoter
CDS: coding sequence
DCMU: 3-(3,4-Dichlorophenyl)-1,1-dimethylurea
ET: enhancer trap FR far red
FRL: far-red light
*GFP*: green fluorescent protein
MS: Murashige and Skoog medium
PCR: polymerase chain reaction
PSII: photosystem II
qRT-PCR: quantitative real-time PCR
RL: red light
RUBISCO: ribulose-1,5-bisphosphate carboxylase/oxygenease
SD: synthetic dropout
*SIG5*: sigma factor 5
TAIL-PCR: thermal asymmetric interlaced PCR
UAS: upstream activation sequence
UTR: untranslated region
Y1H: yeast-one-hybrid

## References

[1] Terzaghi W.B., Cashmore A.R. (1995). Light-regulated transcription. Annu. Rev. Plant Physiol. Plant Mol. Biol..

[2] Troxler R.F., Zhang F., Hu J., Bogorad L. (1994). Evidence that sigma factors are components of chloroplast RNA polymerase. Plant Physiol..

[3] Block A., Dangl J.L., Hahlbrock K., Schulze-Lefert P. (1990). Functional borders, genetic fine-structure, and distance requirements of cis elements mediating light responsiveness of the parsley chalcone cynthase promoter. Proc. Natl. Acad. Sci. USA.

[4] Gilmartin P.M., Sarokin L., Memelink J., Chua N.-H. (1990). Molecular light switches for plant genes. Plant Cell.

[5] Giuliano G., Pichersky E., Malik V.S., Timko M.P., Scolnik P.A., Cashmore A.R. (1988). An evolutionarily conserved protein-binding sequence upstream of a plant light-regulated gene. Proc. Natl Acad. Sci. USA.

[6] Ha S.B., An G.H. (1988). Identification of upstream regulatory elements involved in the developmental expression of the *Arabidopsis thaliana cab1* gene. Proc. Natl. Acad. Sci. USA.

[7] Borello U., Ceccarelli E., Giuliano G. (1993). Constitutive, light-responsive and circadian clock-responsive factors compete for the different L-box elements in plant light-regulated promoters. Plant J..

[8] Grob U., Stuber K. (1987). Discrimination of phytochrome dependent light inducible from non-light inducible plant genes—Prediction of a common light-responsive element (Lre) in phytochrome dependent light inducible plant genes. Nucleic Acids Res..

[9] Srivastava R., Rai K.M., Srivastava M., Kumar V., Pandey B., Singh S.P., Bag S.K., Singh B.D., Tuli R., Sawant S.V. (2014). Distinct role of core promoter architecture in regulation of light-mediated responses in plant genes. Mol. Plant.

[10] Martinez-Garcia J.F., Huq E., Quail P.H. (2000). Direct targeting of light signals to a promoter element-bound transcription factor. Science.

[11] Rüdiger W., Oster U., Eaton-Rye J.J., Tripathy B.C., Sharkey T.D. (2012). Intracellular signaling from plastids to the nucleus. Photosynthesis.

[12] Chory J. (2010). Light signal transduction: An infinite spectrum of possibilities. Plant J..

[13] Nelson T., Harpster M.H., Mayfield S.P., Taylor W.C. (1984). Light-regulated gene expression during maize leaf development. J. Cell Biol..

[14] Lorrain S., Genould T., Fankhauser C. (2006). Let there be light in the nucleus!. Curr. Opin. Plant Biol..

[15] Millar A.J. (2004). Input signals to the plant circadian clock. J. Exp. Bot..

[16] Sullivan J.A., Deng X.W. (2003). From seed to seed: The role of photoreceptors in *Arabidopsis* development. Dev. Biol..

[17] Larkin R.M., Ruckle M.E. (2008). Integration of light and plastid signals. Curr. Opin. Plant Biol..

[18] Neff M.M., Chory J. (1998). Genetic Interactions between phytochrome A, phytochrome B, and cryptochrome 1 during *Arabidopsis* development. Plant Physiol..

[19] Chen F., Li B., Li G., Charron J.B., Dai M., Shi X., Deng X.W. (2014). Arabidopsis phytochrome A directly targets numerous promoters for individualized modulation of genes in a wide range of pathways. Plant Cell.

[20] Haseloff J. (1999). *GFP* variants for multispectral imaging of living cells. Methods Cell Biol..

[21] Laplaze L., Parizot B., Baker A., Ricaud L., Martiniére A., Auguy F., Franche C., Nussaume L., Bogusz D., Haseloff J. (2005). GAL4-*GFP* enhancer trap lines for genetic manipulation of lateral root development in *Arabidopsis thaliana*. J. Exp. Bot..

[22] He Y.H., Tang W.N., Swain J.D., Green A.L., Jack T.P., Gan S.S. (2001). Networking senescence-regulating pathways by using Arabidopsis enhancer trap lines. Plant Physiol..

[23] Gardner M.J., Baker A.J., Assie J.M., Poethig R.S., Haseloff J.P., Webb A.A.R. (2009). GAL4 *GFP* enhancer trap lines for analysis of stomatal guard cell development and gene expression. J. Exp. Bot..

[24] Tsunoyama Y., Ishizaki Y., Morikawa K., Kobori M., Nakahira Y., Takeba G., Toyoshima Y., Shiina T. (2004). Blue light-induced transcription of plastid-encoded *psbD* gene is mediated by a nuclear-encoded transcription initiation factor, At*SIG5*. Proc. Natl. Acad. Sci. USA.

[25] Noordally Z.B., Ishii K., Atkins K.A., Wetherill S.J., Kusakina J., Walton E.J., Kato M., Azuma M., Tanaka K., Hanaoka M. (2013). Circadian control of chloroplast transcription by a nuclear-encoded timing signal. Science.

[26] Pena-Ahumada A., Kahmann U., Dietz K.J., Baier M. (2006). Regulation of peroxiredoxin expression versus expression of Halliwell-Asada-Cycle enzymes during early seedling development of *Arabidopsis thaliana*. Photosyn. Res..

[27] Liu Y.G., Mitsukawa N., Oosumi T., Whittier R.F. (1995). Efficient isolation and mapping of *Arabidopsis thaliana* T-DNA insert junctions by thermal asymmetric interlaced PCR. Plant J..

[28] Winter D., Vinegar B., Nahal H., Ammar R., Wilson G.V., Provart N.J. (2007). An “Electronic fluorescence pistograph” browser for exploring and analyzing large-scale data sets. PLoS One.

[29] Mochizuki T., Onda Y., Fujiwara E., Wada M., Toyoshima Y. (2004). Two independent light signals cooperate in the activation of the plastid *psbD* blue light-responsive promoter in *Arabidopsis*. FEBS Lett..

[30] Onda Y., Yagi Y., Saito Y., Takenaka N., Toyoshima Y. (2008). Light induction of Arabidopsis *SIG1* and *SIG5* transcripts in mature leaves: Differential roles of cryptochrome 1 and cryptochrome 2 and dual function of *SIG5* in the recognition of plastid promoters. Plant J..

[31] Tsunoyama Y., Morikawa K., Shiina T., Toyoshima Y. (2002). Blue light specific and differential expression of a plastid sigma factor, *SIG5* in *Arabidopsis thaliana*. FEBS Lett..

[32] Nagashima A., Hanaoka M., Shikanai T., Fujiwara M., Kanamaru K., Takahashi H., Tanaka K. (2004). The multiple-stress responsive plastid sigma factor, *SIG5*, directs activation of the *psbD* blue light-responsive promoter (BLRP) in *Arabidopsis thaliana*. Plant Cell Physiol..

[33] Oh S., Montgomery B.L. (2014). Phytochrome-dependent coordinate control of distinct aspects of nuclear and plastid gene expression during anterograde signaling and photomorphogenesis. Front. Plant Sci..

[34] Rombauts S., Déhais P., van Montagu M., Rouzé P. (1999). PlantCARE, a plant *cis*-acting regulatory element database. Nucleic Acids Res..

[35] Higo K., Ugawa Y., Iwamoto M., Korenaga T. (1999). Plant *cis*-acting regulatory DNA elements (PLACE) database. Nucleic Acids Res..

[36] Alonso J.M., Stepanova A.N., Leisse T.J., Kim C.J., Chen H.M., Shinn P., Stevenson D.K., Zimmerman J., Barajas P., Cheuk R. (2003). Genome-wide insertional mutagenesis of *Arabidopsis thaliana*. Science.

[37] Kagawa T., Kasahara M., Abe T., Yoshida S., Wada M. (2004). Function analysis of phototropin2 using fern mutants deficient in blue light-induced chloroplast avoidance movement. Plant Cell Physiol..

[38] Guo H., Duong H., Ma N., Lin C. (1999). The Arabidopsis blue light receptor cryptochrome 2 is a nuclear protein regulated by a blue light-dependent post-transcriptional mechanism. Plant J..

[39] Lopez-Juez E., Dillon E., Magyar Z., Khan S., Hazeldine S., de Jager S.M., Murray J.A.H., Beemster G.T.S., Bogre L., Shanahan H. (2008). Distinct light-initiated gene expression and cell cycle programs in the shoot apex and cotyledons of Arabidopsis. Plant Cell.

[40] Schäfer E., Haupt W., Shropshire W., Mors H. (1983). Blue light effects in phytocrome-mediated responses. Encyclopedia of Plant Physiology.

[41] Muramoto T., Kohchi T., Yokota A., Hwang I.H., Goodman H.M. (1999). The *Arabidopsis* photomorphogenic mutant *hy1* is deficient in phytochrome chromophore biosynthesis as a result of a mutation in a plastid heme oxygenase. Plant Cell.

[42] Oyama T., Shimura Y., Okada K. (1997). The *Arabidopsis* HY5 gene encodes a bZIP protein that regulates stimulus-induced development of root and hypocotyl. Genes Dev..

[43] Chattopadhyay S., Puente P., Deng X.W., Wei N. (1998). Combinatorial interaction of light-responsive elements plays a critical role in determining the response characteristics of light-regulated promoters in *Arabidopsis*. Plant J..

[44] Montemartini M., Kalisz H.M., Hecht H.J., Steinert P., Flohe L. (1999). Activation of active-site cysteine residues in the peroxiredoxin-type tryparedoxin peroxidase of *Crithidia fasciculata*. Eur. J. Biochem..

[45] McKendree W.L., Ferl R.J. (1992). Functional elements of the *Arabidopsis* Adh promoter include the G-box. Plant Mol. Biol..

[46] Donald R.G., Cashmore A.R. (1990). Mutation of either G box or I box sequences profoundly affects expression from the Arabidopsis rbcS-1A promoter. EMBO J..

[47] Laisk A., Oja V., Eichelmann H., Dall’Osto L. (2014). Action spectra of photosystems II and I and quantum yield of photosynthesis in leaves in State 1. Biochim. Biophys. Acta.

[48] Pfannschmidt T., Nilsson A., Allen J.F. (1999). Photosysnthetic control of chloroplast gene expression. Nature.

[49] Trebst A., Depka B., Kraft B., Johanningmeier U. (1988). The Q_B_ site modulates the conformation of the photosystem II reaction center polypeptides. Photosyn. Res..

[50] Rook F., Corke F., Baier M., Holman R., May A.G., Bevan M.W. (2006). *Impaired sucrose induction1* encodes a conserved plant-specific protein that couples carbohydrate availability to gene expression and plant growth. Plant J..

[51] NCBI-Server. http://www.ncbi.nlm.nih.gov.

[52] Sheffield J.B. (2007). ImageJ, a useful tool for biological image processing and analysis. Microsc. Microanal..

[53] Porra R.J., Thompson W.A., Kriedemann P.E. (1989). Determination of accurate extinction coefficients and simultaneous equations for assaying chlorophyll-a and chlorophyll-b extracted with four different solvents: Verification of the concentration of chlorophyll standards by atomic absorption spectroscopy. Biochim. Biophys. Acta.

[54] Baier M., Ströher E., Dietz K.J. (2004). The acceptor availability at photosystem I and ABA control nuclear expression of 2-Cys peroxiredoxin-A in *Arabidopsis thaliana*. Plant Cell Physiol..

[55] Arvidsson S., Kwasniewski M., Riano-Pachon D.M., Mueller-Roeber B. (2008). QuantPrime—A flexible tool for reliable high-throughput primer design for quantitative PCR. BMC Bioinform..

[56] Quantprime. http://www.quantprime.mpimp-golm.mpg.de.

[57] Bustin S.A., Benes V., Garson J.A., Hellemans J., Huggett J., Kubista M., Mueller R., Nolan T., Pfaffl M.W., Shipley G.L. (2009). The MIQE guidelines: Minimum information for publication of quantitative real-time PCR experiments. Clin. Chem..

[58] An Y.Q., McDowell J.M., Huang S., McKinney E.C., Chambliss S., Meagher R.B. (1996). Strong, constitutive expression of the *Arabidopsis* ACT2/ACT8 actin subclass in vegetative tissues. Plant J..

[59] Pfaffl M.W. (2001). A new mathematical model for relative quantification in real-time RT-PCR. Nucleic Acids Res..

[60] Ross J. (1995). Messenger-RNA stability in mammalian cells. Microbiol. Rev..

[61] Gutierrez R.A., Ewing R.M., Cherry J.M., Green P.J. (2002). Identification of unstable transcripts in *Arabidopsis* by cDNA microarray analysis: Rapid decay is associated with a group of touch- and specific clock-controlled genes. Proc. Natl. Acad. Sci. USA.

[62] Karimi M., Inze D., Depicker A. (2002). GATEWAY((TM)) vectors for *Agrobacterium*-mediated plant transformation. TIPS.

[63] Koncz C., Schell J. (1986). The promoter of TL-DNA gene *5* controls the tissue-specific expression of chimaeric genes carried by a novel type of *Agrobacterium* binary vector. Mol. Gen. Genet..

[64] Weigel D., Glazebrook J. (2002). Arabidopsis: A Laboratory Manual.

[65] Voinnet O., Rivas S., Mestre P., Baulcombe D. (2003). An enhanced transient expression system in plants based on suppression of gene silencing by the p19 protein of tomato bushy stunt virus. Plant J..

[66] English J.J., Davenport G.F., Elmayan T., Vaucheret H., Baulcombe D.C. (1997). Requirement of sense transcription for homology-dependent virus resistance and trans-inactivation. Plant J..

[67] PLACE. http://dna.affrc.go.jp/PLACE/signal-scan.html.

[68] PlantCARE. http://bioinformatics.psb.ugent.be/web-tools/plantcare/html.

[69] Gietz R.D., Schiestl R.H. (2007). High-efficiency yeast transformation using the LiAc/SS carrier DNA/PEG method. Nat. Protoc..

[70] Lascéve G., Leymarie J., Olney M.A., Liscum E., Christie J.M., Vavasseur A., Briggs W.R. (1999). *Arabidopsis* contains at least four independent blue-light-activated signal transduction pathways. Plant Physiol..

[71] (2009). LSM Image Browser Software.

[72] Schreiber U., Bilger W. (1993). Progress in chlorophyll fluorescence research: Major developments during the past years in retrospect. Prog. Bot..

[73] Tanaka K., Tozawa Y., Mochizuki N., Shinozaki K., Nagatani A., Wakasa K., Takahashi H. (1997). Characterization of three cDNA species encoding plastid RNA polymerase sigma factors in *Arabidopsis thaliana*: Evidence for the sigma factor heterogeneity in higher plant plastids. FEBS Lett..

[74] Fujiwara M., Nagashima A., Kanamaru K., Tanaka K., Takahashi H. (2000). Three new nuclear genes, *sigD*, *sigE* and *sigF*, encoding putative plastid RNA polymerase [sigma] factors in *Arabidopsis thaliana*. FEBS Lett..

[75] Sugiura M. (1989). The Chloroplast Chromosomes in Land Plants. Annu. Rev. Cell. Biol..

[76] Hoffer P.H., Christopher D.A. (1997). Structure and blue-light-responsive transcription of a chloroplast *psbD* promoter from *Arabidopsis thaliana*. Plant Physiol..

[77] Thum K.E., Kim M., Morishige D.T., Eibl C., Koop H.U., Mullet J.E. (2001). Analysis of barley chloroplast *psbD* light-responsive promoter elements in transplastomic tobacco. Plant Mol. Biol..

[78] Kanamaru K., Nagashima A., Fujiwara M., Shimada H., Shirano Y., Nakabayashi K., Shibata D., Tanaka K., Takahashi H. (2001). An *Arabidopsis* sigma factor (*SIG2*)-dependent expression of plastid-encoded tRNAs in chloroplasts. Plant Cell Physiol..

[79] Ishizaki Y., Ozono K., Takenaka C., Tsunoyama Y., Nakahira Y., Tanaka K., Kanamaru K., Hanaoka M., Shiina T. (2006). An *Arabidopsis* double mutant *sig2sig6* exhibits albino phenotype. Plant Cell Physiol..

[80] Shalitin D., Yu X., Maymon M., Mockler T., Lin C. (2003). Blue light-dependent *in vivo* and *in vitro* phosphorylation of *Arabidopsis* cryptochrome 1. Plant Cell.

[81] Shalitin D., Yang H., Mockler T.C., Maymon M., Guo H., Whitelam G.C., Lin C. (2002). Regulation of *Arabidopsis* cryptochrome 2 by blue-light-dependent phosphorylation. Nature.

[82] Sharrock R.A., Clack T. (2002). Patterns of expression and normalized levels of the five *Arabidopsis* phytochromes. Plant Physiol..

[83] Lee J., He K., Stolc V., Lee H., Figueroa P., Gao Y., Tongprasit W., Zhao H., Lee I., Deng X.W. (2007). Analysis of transcription factor HY5 genomic binding sites revealed its hierarchical role in light regulation of development. Plant Cell.

[84] Pfannschmidt T., Nilsson A., Tullberg A., Link G., Allen J.F. (1999). Direct transcriptional control of the chloroplast genes *psbA* and *psaAB* adjusts photosynthesis to light energy distribution in plants. IUBMB Life.

[85] Tullberg A., Alexciev K., Pfannschmidt T., Allen J.F. (2000). Photosynthetic electron flow regulates transcription of the *psaB* gene in pea (*Pisum sativum* L.) chloroplasts through the redox state of the plastoquinone pool. Plant Cell Physiol..

[86] Koch K.E. (1999). Carbohydrate-modulated gene expression in plants. Annu. Rev. Plant Physiol. Plant Mol. Biol..

[87] Allen J.F., Pfannschmidt T. (2000). Balancing the two photosystems: Photosynthetic electron transfer governs transcription of reaction centre genes in chloroplasts. Philos. Trans. R. Soc. Lond. B.

[88] Tikkanen M., Gollan P.J., Suorsa M., Kangasjarvi S., Aro E.M. (2012). STN7 operates in retrograde signaling through controlling redox balance in the electron transfer chain. Front. Plant Sci..

[89] Aro E.M., Virgin I., Andersson B. (1993). Photoinhibition of Photosystem II. Inactivation, protein damage and turnover. Biochim. Biophys. Acta.

[90] Keren N., Berg A., van Kan P.J., Levanon H., Ohad I. (1997). Mechanism of photosystem II photoinactivation and D1 protein degradation at low light: The role of back electron flow. Proc. Natl. Acad. Sci. USA.

